# Experiences with telemedicine for HIV care in two federally qualified health centers in Los Angeles: a qualitative study

**DOI:** 10.1186/s12913-023-09107-1

**Published:** 2023-02-15

**Authors:** Daisy Walker, Corrina Moucheraud, Derrick Butler, Jerome de Vente, Kevin Tangonan, Steven Shoptaw, Judith S. Currier, Jay Gladstein, Risa Hoffman

**Affiliations:** 1 Division of Infectious Diseases, David Geffen School of Medicine at UCLA, Los Angeles California, USA; 2 Department of Health Policy and Management, Fielding School of Public Health at UCLA, Los Angeles California, USA; 3 To Help Everyone, Los Angeles California, USA; 4 APLA Health, Los Angeles California, USA; 5 Department of Family Medicine, David Geffen School of Medicine at UCLA, Los Angeles California, USA

**Keywords:** HIV, Telemedicine, Qualitative, Benefits, Challenges

## Abstract

**Background:**

The SARS-CoV-2 pandemic has resulted in an increase in telemedicine utilization for routine HIV care. However, there is limited information on perceptions of and experiences with telemedicine from United States (U.S.) federally qualified health centers (FQHCs) offering HIV care. We sought to understand telemedicine experiences of stakeholders with various roles: people living with HIV (PLHIV), clinical (clinicians and case managers), programmatic (clinic administrators), and policy (policymakers).

**Methods:**

Qualitative interviews about benefits and challenges of telemedicine (telephone and video) for HIV care were conducted with 31 PLHIV and 23 other stakeholders (clinicians, case managers, clinic administrators, and policymakers). Interviews were transcribed, translated to English if conducted in Spanish, coded, and analyzed for major themes.

**Results:**

Almost all PLHIV felt capable of engaging in telephone visits, with some expressing interest in learning how to use video visits as well. Nearly all PLHIV wanted to continue telemedicine as part of their routine HIV care, and this was also endorsed by clinical, programmatic and policy stakeholders. Interviewees agreed that telemedicine for HIV care has benefits for PLHIV, especially savings of time and transportation costs, which also reduced stress. Clinical, programmatic, and policy stakeholders expressed concerns around patients’ technological literacy and resources, as well as their access to privacy, and some felt that PLHIV strongly preferred in-person visits. These stakeholders also commonly reported clinic-level implementation challenges, including integrating telephone and video telemedicine into workflows and difficulty with video visit platforms.

**Conclusions:**

Telemedicine for HIV care, largely delivered via telephone (audio-only), was highly acceptable and feasible for both PLHIV, clinicians, and other stakeholders. Addressing barriers for stakeholders in incorporating video visits will be important for the successful implementation of telemedicine with video as part of routine HIV care at FQHCs.

## Background

The severe acute respiratory syndrome coronavirus 2 (SARS-CoV-2) pandemic and resultant widespread stay-at-home orders in early 2020 catapulted telemedicine to the mainstream for health care in the United States [1] and introduced telemedicine to a broader range of care settings, including federally qualified health centers (FQHCs). Beginning in March 2020, the Centers for Medicare & Medicaid Services (CMS) provided pandemic-related regulatory waivers that allowed clinics to offer telephone (audio only) and video visits to beneficiaries, with compensation equivalent to in-person care [2].

Prior to the pandemic, telemedicine was widely documented as an effective supplement to in-person care for certain chronic medical conditions, such as mental health, [3] diabetes, [4] and cardiovascular diseases [5]. Telemedicine for these conditions has been associated with equivalent or better outcomes than in-person care, as well as reduced costs for patients and for health systems [6–8]. However, telemedicine has received significantly less attention as a strategy for delivering HIV care, particularly within public health systems, which care for individuals who have been historically marginalized and face significant barriers to care. A small number of studies from the U.S. (conducted within University-affiliated and Veterans Affairs health systems) and Canada suggest that telemedicine could have many benefits when used for HIV care, including improved access to care and timeliness of care, [9] ease of medication reconciliation when patients are at home for telemedicine visits, [10] and improvements in viral suppression [11].

The degree to which telemedicine benefits apply to people with HIV cared for at FQHCs is not well-established, despite pandemic-related scale-up in these settings. According to a national survey of clinics funded by the Ryan White program (RW), the largest federal program providing care and services for people living with HIV (PLHIV), nearly all RW clinics (99%) were offering telemedicine as of September 2020 in comparison to only 22% prior to the SARS-CoV-2 pandemic [12]. FQHCs represent a unique context to understand factors that impact telemedicine use, both in terms of the resources available at clinics and their patient population. FQHCs often have smaller IT departments, less sophisticated electronic medical record systems lacking functional or integrated telemedicine capabilities, and greater staffing constraints as compared to private and academic clinical settings. We therefore sought to understand the experiences of stakeholders with various roles including PLHIV, clinical (clinicians and case managers), programmatic (clinic administrators), and policy (policymakers), with a focus on benefits, barriers, and opportunities for improvement of telemedicine in FQHCs.

## Methods

### Setting

From March to July 2021, we conducted in-depth interviews (IDIs) with stakeholders from two FQHCs located in south Los Angeles, within the county’s Service Planning Area (SPA) 6. SPA 6 has the second-highest rate of new HIV infections in the county and a below-average viral suppression rate of 59% [13]. These two health centers serve underinsured individuals with high levels of unemployment, housing instability, substance use, and mental health disorders.

Once the initial stay-at-home orders were announced in March 2020, both clinics reduced the number of patients coming for care in-person and allowed care to be delivered via telemedicine visits (predominantly telephone-based visits). Patients who came to the clinic in person did so only if telemedicine (telephone or video) was declined or was not an option for their care, such as the need for a physical exam for diagnostic purposes, for routine or urgent bloodwork, or for other specific primary care that required monitoring that could not be achieved remotely (blood pressure monitoring and blood glucose monitoring if the patient was unable to do this at home). As SARS-CoV-2 case numbers ebbed and flowed with each surge, clinics’ use of telemedicine visits mirrored these patterns.

### Sample

PLHIV participants were selected from both sites using convenience sampling. Patients were eligible to participate if they were at least 18 years old, had been receiving HIV care at the particular clinic for at least 12 months, and consented to doing the interview when asked by their provider during either an in-person or telemedicine visit. We decided to include people who had not used telephone or video telemedicine to capture their unique barriers to this type of care, perspectives on use of telemedicine, and openness to use in the future. Understanding why people may not have utilized telemedicine, particularly during the pandemic, is important to determine whether interventions could be tailored to this group to improve their uptake in the future. We also conducted IDIs with clinical and programmatic stakeholders at the two clinics, including clinicians, case managers, and clinic administrators involved in service delivery for PLHIV, as well as policy stakeholders from Los Angeles County with roles as policymakers in county-wide HIV and public health initiatives.

### Interview guide development

Three interview guides were developed separately: one for PLHIV, one for clinicians and case managers (patient-facing roles), and one for clinic administrators and policymakers. Guides were designed to understand perspectives on telemedicine for HIV care. The guide for PLHIV was informed by the Modified Framework of Access, with questions focusing on access to technological resources needed for telemedicine (i.e. smartphones, computers, Wi-Fi or mobile data), personal use and perceptions of telemedicine, and comparisons between telemedicine and in-person visits [14]. The guide for clinical stakeholders and the guide for programmatic and policy stakeholders were both informed by the Consolidated Framework for Implementation Research, with questions focusing on implementation of telemedicine for HIV care, system-level barriers and enablers of telemedicine, and perceptions of PLHIV's experience with telemedicine [15]. All interview guides began with a set of survey questions to determine participants’ use of telemedicine (both telephone and video), followed by a core set of qualitative interview questions about telemedicine (open-ended), and ended with a final set of survey questions on participants’ sociodemographic characteristics, which were closed (survey) questions. For PLHIV, these survey questions asked their age, gender, race, employment status, years on ART, housing status, number of in-person and telemedicine HIV visits in the past year, and use of telemedicine for other types of health care. Clinical, programmatic, and policy stakeholders were asked their age, gender, professional role, and years of experience for HIV care and for telemedicine.

### Data collection

IDIs were conducted over telephone or Zoom (based on participant preference) to promote the safety of research staff and study participants during the SARS-CoV-2 pandemic. Interviews ranged from 20 to 75 min and were conducted by four research team members in English or Spanish, depending on the study participant’s language of choice. Each interview was audio recorded after obtaining oral consent from participants, and compensation for interview completion was provided ($40 cash for PLHIV, available for pick-up at the clinics, and a $40 gift card, with the code emailed, for clinical, programmatic, and policy stakeholders). The study was approved by the Institutional Review Board at the University of California, Los Angeles (IRB #20–001508).

### Data analysis

Audio recordings were transcribed and, for interviews that were conducted in Spanish, translated to English. We developed two preliminary codebooks, one for interviews conducted with PLHIV, based on the Modified Framework of Access; [14] and another for interviews conducted with clinical, programmatic, and policy stakeholders, based on the Consolidated Framework for Implementation Research [15]. Each codebook was piloted by two members of the study team: each study member independently reviewed and coded three transcripts and, after multiple rounds of consultation and revisions, the team agreed on a codebook. The transcripts were then double coded utilizing Dedoose software. Conflicts were resolved by a tiebreaker if consensus could not be reached between the coders.

We used summary statistics to describe participant clinical and demographic data. For qualitative data, we performed thematic analysis with three coauthors. First, we reviewed all coded extracts to identify where codes were connected, i.e. themes, utilizing Braun and Clarke’s definition of themes as “represent[ing] some level of patterned response or meaning within the data set.” [16] For example, our codebook for data from PLHIV included the codes “travel time”, “transportation costs”, “opportunity costs” and “wait time.” During analysis, we found that these codes were applied to the same or overlapping blocks of text – suggesting that PLHIV were discussing concepts intrinsically related to one another – so, we created a theme titled “time and costs associated with the care model” to clarify the relationship between the time-related concepts and the cost-related concepts. We repeated this process until all codes were represented within themes. We ensured that themes did not substantively overlap. We then analyzed differences within themes by specific respondent characteristics, including age and duration on antiretroviral therapy (ART). We defined older PLHIV as greater than the median age for the population and younger as less than the median age. Likewise, we defined longer duration on ART as greater than the median years on ART for the population and shorter as less than the median years. Finally, we represented prevalence of participant responses within our qualitative analysis using descriptors such as “most,” “many,” and “a number of” – in line with conventions established in the literature [17–19].

## Results

### PLHIV

The median age of PLHIV completing interviews was 50 years (range 23–65). Twenty-one PLHIV (68%) identified as cisgender men, seven (22%) as cisgender women, and three (10%) as transgender women (Table 1). The majority of PLHIV identified as either Hispanic/Latino/a or Black/African-American (65%), with eleven (36%) identifying as Hispanic or Latino/a and nine (29%) identifying as Black or African-American. Most PLHIV were English-speaking (*N* = 24, 78%), with the remainder (*N* = 7, 22%) reporting Spanish as their primary language. Twenty-eight PLHIV (90%) had used telemedicine as part of their HIV care in the past year, and 24 of these PLHIV (86%) reported that their visits had been conducted over telephone only. Characteristics of telemedicine use are described in Table 2.

**Table 1:** Demographic Characteristics of PLHIV

	**Overall** **(*N* = 31)**	**Clinic 1** **(*N* = 15)**	**Clinic 2** **(*N* = 16)**
**Age**, Median (IQR)	50 (40–58)	50 (32–57)	51.5 (42.5–58.5)
**Gender**, N (%)			
Cisgender female	7 (22)	2 (13)	5 (31)
Cisgender male	21 (68)	10 (67)	11 (69)
Transgender female	3 (10)	3 (20)	0 (0)
**Race/Ethnicity**, N (%)			
Black or African-American	9 (29)	1 (7)	8 (50)
Hispanic or Latino/a	11 (36)	5 (33)	6 (37)
Native American	1 (3)	1 (7)	0 (0)
White/Caucasian	4 (13)	3 (20)	1 (6)
Multi-racial	6 (19)	5 (33)	1 (6)
**Primary language**, N (%)			
English	24 (78)	13 (87)	11 (69)
Spanish	7 (22)	2 (13)	5 (31)
**Years on ART**, Median (IQR)	8 (3–20)	8 (3–23)	8 (3–15)
**Employment status**, N (%)^a^			
Working	12 (39)	6 (40)	6 (37)
Not working	18 (58)	8 (53)	10 (63)
**Stable housing**, N (%)^a^			
Yes	27 (87)	13 (87)	14 (87)
No	3 (10)	1 (7)	2 (13)

^a^Missing data (*N*=1): One participant was missing data for employment status and stable housing

**Table 2:** Use of Telemedicine by PLHIV

	**Overall** **(*N* = 31)** N (%)	**Clinic 1** **(*N* = 15)** N (%)	**Clinic 2** **(*N* = 16)** N (%)
**Used telemedicine for HIV care in past year**			
Yes	28 (90)	12 (80)	16 (100)
No	3 (10)	3 (20)	0 (0)
**Mode of telemedicine used**			
Telephone only	24 (78)	8 (53)	16 (100)
Video only	2 (6)	2 (13)	0 (0)
Both telephone and video	2 (6)	2 (13)	0 (0)
N/A (No use of telemedicine in past year)	3 (10)	3 (20)	0 (0)
**Technological resources available**			
Phone (no internet)	1 (3)	0 (0)	1 (6)
Smartphone (with internet)	7 (22)	3 (20)	4 (25)
Smartphone and computer	23 (75)	12 (80)	11 (69)
**Number of in-person visits in prior year for HIV care**^a^			
0	0 (0)	0 (0)	0 (0)
1–2	7 (22)	3 (20)	4 (25)
3–4	11 (36)	4 (27)	7 (44)
5 or more	13 (42)	8 (53)	5 (31)
**Number of telemedicine visits in prior year for HIV care**^a^			
0	3 (10)	3 (20)	0 (0)
1–2	14 (45)	7 (47)	7 (44)
3–4	12 (39)	5 (33)	7 (44)
5 or more	2 (6)	0 (0)	2 (13)
**Use of telemedicine for other non-HIV care**^**b**^			
Yes	11 (36)	10 (67)	1 (6)
No	20 (64)	5 (33)	15 (94)

^a^Based on self-report, ^b^Types of non−HIV care utilized via telemedicine: mental health, case management, SARS−CoV−2 care, specialized care (oncology, nephrology, physical therapy, dentistry)

The following themes emerged during thematic analysis of PLHIV interviews, which are addressed in detail below: time and costs associated with the care model; resources for telemedicine and technological literacy; access to privacy for telemedicine; impact of telemedicine on the interpersonal dynamic; interest in future telemedicine; and interest in video modality of telemedicine.

#### Time and costs associated with the care model

When asked about time spent seeking care, most PLHIV reported that using telemedicine (predominantly telephone) for their HIV care saved significant time because they did not need to commute to and from the clinic – *“by telephone, you are at home…It saves you the time of going, the time of driving, whether you're going by bus, train, or your own transportation. It saves you time right there.”* (Cisgender female, 59 years old, 2 years on ART)

Telemedicine visits also saved time spent waiting for the clinician. While participants did sometimes report a wait time for telemedicine appointments, this was shorter than for an in-person appointment. Respondents citing this benefit were mostly those who had been on ART for longer. One participant shared,* “I don’t have a car so I need to take public transportation and it’s a bit over one hour commute. Plus, the wait time in reception, and the doctor, that takes an additional two or three hours—which I am saving now because telemedicine is coming to me and I don’t have to invest all that time.”* (Cisgender male, 58 years old, 14 years on ART) Many PLHIV also shared that telemedicine saved money on transportation costs, including for public transportation, gas for a personal vehicle, or a rideshare such as Uber or Lyft. This was especially beneficial for patients who lived far from the clinic because, as one participant explained, *“Going in person—it expends a lot of time, energy, money that I don’t really have because I’m up here in [North County] and their clinic is down in L.A. It’s a drive and I don’t have $200, $300 a month to throw away on gas.”* (Cisgender male, 56 years old, 20 years on ART)

Some PLHIV also appreciated that telemedicine did not require taking any or as much time off work, and therefore resulted in fewer earnings losses as compared to in-person visits, stating,* “[Telemedicine] saves me time, I don’t go to the clinic, I don’t have to ask for time off at work: ‘Tomorrow I’m going to come two hours later, I have an appointment,’ and so on.”* (Cisgender male, 45 years old, 9 years on ART)

#### Resources for telemedicine and technological literacy

When PLHIV were asked about their ability to utilize telemedicine for HIV care, many felt able to successfully connect and engage in a telemedicine visit via telephone. While almost all (*N* = 30, 97%) PLHIV reported having access to either a smartphone, computer and/or tablet, as well as either mobile data and/or Wi-Fi, a few without access to these technological resources expressed frustration with their lack of access and desired better technology for telemedicine visits with video. This sentiment is shared by one study participant who desired more resources such as *“access to a computer, or a tablet, or something. I don’t know. Because this phone thing is not really working for me.”* (Cisgender male, 50 years old, 10 years on ART) Few PLHIV had experience with video visits; however, those that did reported few to no barriers. Some PLHIV reported experience with video visits for their mental health care, or video calls with their family and friends during the pandemic, and shared that there was a learning curve, but they quickly became confident in their ability to use video for these types of communication, with one participant stating that it *“…wasn’t hard. All you did was you get this new app and then they send you a notification and that’s just a link, so it’s real simple.”* (Cisgender male, 56 years old, 20 years on ART)

#### Access to privacy for telemedicine

PLHIV were asked if they had privacy for either telephone or video telemedicine visits. Many—particularly older interviewees—had consistent access to privacy in their household. As one participant expressed*: “If I needed to do a Zoom call or a video conferencing, I will do it in my apartment because I live alone, so it is private.”* (Cisgender male, 56 years old, 8 years on ART) Even though they had privacy at their homes, a few PLHIV liked that telemedicine might give them the opportunity to attend their visits in another location, describing that,* “I can be at the beach, or I can be at a park. I can probably have a phone call in a location like that. It would really be nice to actually be out there. Normally, I’m at home, where it’s more quiet.”* (Cisgender male, 34 years old, 5 years on ART) However, some PLHIV, particularly those who had not disclosed their HIV status and lived in shared housing, said that a lack of privacy was a major barrier for accessing telephone or video telemedicine for their HIV care, with one participant stating, *“I'm in a transitional living home. Privacy's a big one. That's a big emphasis as to why I would prefer to go inside the clinic.”* (Transgender female, 26 years old, 5 years on ART)

#### Impact of telemedicine on the interpersonal dynamic

Most PLHIV reported that their relationship and interpersonal dynamic with their clinician was unaffected by the use of telephone and/or video telemedicine. Those with long-term clinician relationships felt they had established comfort and trust, which continued when communicating over the telephone or video. These respondents tended to be older and in care for longer, but some recently-diagnosed PLHIV reported this as well, with participants sharing*, “Well, for over the phone, I’m comfortable to be honest with you. I’m comfortable either way’cause I’ve been going there for so long, they’re like my family.”* (Cisgender female, 47 years old, 28 years on ART) Another participant agreed and said, *“[Doctor’s name] has really been amazing. [Doctor’s name] is perfect for telehealth, because I feel like I pick up the pieces where we left off in person, and it doesn't feel impersonal. It doesn't feel distant, it doesn't feel—it feels great actually.”* (Cisgender male, 57 years old, 1 year on ART)

Many PLHIV said they had felt uncertain about telemedicine because they had never used it before—but, over time, they became more assured of telemedicine’s benefits for their HIV care and confident in their clinician’s ability to assess their needs as they would in-person, as illustrated by this participant: *“I was real apprehensive at first, like, Hmm, am I really gonna get the care needed in my situation with these services? The answer was yes. We still were able to cover everything that’s affecting me and be able to do something about it just as if I was standing right there in the office.”* (Cisgender male, 43 years old, 10 years on ART)

However, some PLHIV preferred the dynamic and connection offered by in-person visits, despite also feeling that telemedicine was an effective form of care, because *“[telemedicine] is good in the sense of it works and it gets the job done. I like it. I think it's better than nothing, but I'm an in-person kind of guy. I like that connection.”* (Cisgender male, 29 years old, 1 year on ART)

When discussing sensitive topics, such as sexually transmitted infections, relationship issues, or substance use, many PLHIV felt equally comfortable with telemedicine (either telephone or video) and in-person care due to their positive relationship with their clinician, sharing *“I also trust [my clinician] to talk about [sensitive topics] over the phone and in person. It doesn’t matter.”* (Transgender female, 60 years old, 26 years on ART) Others preferred telemedicine for these sensitive discussions because they found comfort in the physical distance, reasoning that *“in person, you might not dare to speak about certain things. Over the phone it is easier to say things because the person is not there in front of you.”* (Cisgender male, 58 years old, 14 years on ART) Other respondents said they liked discussing sensitive topics in person and would share more information in person as compared to by telemedicine, such as this participant who stated, *“I like to talk to [my doctor] face to face when it comes to [my personal life and my relationship]… To be there and to have eye contact with her, she can see what else is going on with me… She can tell when I’m depressed…in person is better when I’m talking personal information to her.”* (Cisgender female, 47 years old, 28 years on ART)

With regard to behavioral care and case management, PLHIV tended to feel positively about telephone or video telemedicine’s use for this care and enjoyed their experiences: *“I love [telemedicine] for behavioral care. It's just fantastic…just authentically being comfortable being at home.”* (Cisgender female, 57 years old, 3 years on ART) Another participant appreciated the flexibility of telemedicine for urgent issues:* “I have case managers there that I can always call… They’re very good at helping me out, talking with me,’cause they know what’s going on with me, too… I had an issue about a month ago, and I really needed them, and they were able to step in and help me.”* (Cisgender female, 47 years old, 28 years on ART)

PLHIV agreed that newly diagnosed individuals should have in-person visits and that the transition to telemedicine visits should occur only if and when PLHIV and their clinician are comfortable with this change in care, as expressed by one participant who felt that *“For HIV care, at the beginning almost everything will have to be in person, because they have to be checking your [viral] load and all that month after month… Now after a year that I’ve been undetectable…[the doctor] can give you the option that the rest you need can be by telemedicine.”* (Transgender female, 57 years old, 1 year on ART)

#### Interest in future telemedicine

All PLHIV were asked if they would like to continue having telephone and video telemedicine as an ongoing option for their HIV care, and all but one participant responded in the affirmative. Participants gave many reasons why they would like to incorporate telemedicine into their care long-term, including ease of completing appointments and reduced visits to the clinic, because* “It's just easier to have a couple of [in-person] visits a year, and then the rest of the time, you can get care through telemedicine. It's easier for the doctor, easier for the patient.”* (Cisgender female, 57 years old, 3 years on ART) Another participant shared, *“I think [telemedicine is] the future, that’s what awaits us. I don’t think it will be a 100 percent replacement, but it would be very helpful.”* (Cisgender male, 58 years old, 14 years on ART) PLHIV who spoke Spanish as their primary language exhibited similar interest in future telephone and video telemedicine as English-speaking patients, even if their clinician did not speak Spanish, given they felt comfortable with interpretation via telemedicine. One participant expressed,* “I thought [telemedicine] was perfectly good, totally correct. Because in fact, the doctor asked the questions in English and her assistant translated them into Spanish, and that made me feel comfortable.”* (Cisgender male, 58 years old, 11 years on ART)

When PHLIV were asked what their preferred balance of visit types would be, the most common response was half in-person and half telemedicine visits, mixed throughout the year: *“If the patient comes six times in one year, schedule three and three and alternate in-person appointments with telemedicine appointments. That way the lab can be scheduled for the same day you go in person and the next visit the doctor usually just gives you the results and you don’t need to be there in person to obtain them.”* (Cisgender male, 58 years old, 14 years on ART) The remaining PLHIV reported a diverse range of preferences about the balance between in-person and telemedicine. PLHIV who wanted telemedicine incorporated into their HIV care found this modality to be of best utility for visits to discuss lab results or for follow-up of a previous in-person visit, while in-person visits were necessary for doing lab work, collecting vital signs, and performing physical exams, such as one participant who preferred *“to go in person when I need a blood test and when they give me the results they can just call me and that’s it. That would be ideal.”* (Cisgender male, 45 years old, 9 years on ART)

#### Interest in video modality of telemedicine

Although few PHLIV in our study had experience with video telemedicine, some expressed a hypothetical preference for video over telephone visits reporting these would better mimic an in-person visit, reasoning that*, “Although it’s virtual, you’re having a conversation with someone you’re watching, and it’s as if he’s in front of you. And so that gives you a little more comfort and confidence.”* (Transgender female, 57 years old, 1 year on ART) A few PLHIV were not interested in utilizing video telemedicine as they felt that telephone visits were sufficient and perceived the training needed for video visits as being too burdensome. One participant reported being *“… quite happy with a telephone call. I don’t want to FaceTime. A telephone call does the very same thing… It’s just not me. I’ve never done it, and I don’t intend to start.” *(Cisgender male, 63 years old, 3 years on ART)

### Results: clinical, programmatic, and policy stakeholders

Twenty-three individuals completed an interview, including ten clinicians (four of whom also had leadership roles), four case managers, and six individuals in clinic administrative roles. The remaining three individuals were policymakers involved in HIV and public health initiatives at the county level. Participants had worked in the HIV field for a range of one to thirty-one years and all had their first exposure to telemedicine for HIV care with the onset of the SARS-CoV-2 pandemic stay-at-home orders in March 2020. Two clinicians had prior experience with video telemedicine for other types of care. Participant characteristics are summarized in Table 3.

**Table 3:** Demographic Characteristics of Clinical, Programmatic, and Policy Stakeholders

	**Overall** **(*N*** **= 23)**	**Clinic 1** **(*N*** **= 12)**	**Clinic 2** **(*N*** **= 8)**
**Role**, N (%)			
Clinician (physician or nurse)	10 (43)	7 (58)	3 (38)
Case manager	4 (17)	1 (9)	3 (38)
Clinic administrator	6 (26)	4 (33)	2 (25)
Policymaker	3 (14)	N/A	N/A
**Years working in field of HIV**, Median (IQR)	10 (3–16)	10 (2.5–19.5)	10.5 (4.125–15.5)
*For clinicians and case managers:*	*Total* *(N* = *14)*	*Clinic 1* *(N* = *8)*	*Clinic 2* *(N* = *6)*
**Years providing telemedicine for HIV care**, Median (IQR)	1 (0.83–1.17)	1 (1–1.21)	1 (0.79–1.25)
**Experience providing telemedicine for non-HIV care**, N (%)^a^ ^b^			
Yes	2 (14)	2 (25)	0 (0)
No	11 (79)	5 (63)	6 (100)

^a^Other types of care provided via telemedicine: primary care, mental health; ^b^Missing data (*N*=1)

The following themes emerged from thematic analysis of clinical, programmatic, and policy stakeholder interviews, which are addressed in detail below: perceived impact of telemedicine on patient-level barriers to care; telemedicine implementation: challenges and solutions; telemedicine training; and beliefs about telemedicine.

#### Perceived impact of telemedicine on patient-level barriers to care

Many respondents mentioned that visit attendance was improved with telephone telemedicine, as one clinician stated: *“Our no-show rate [is] actually dropping a little bit with telephone visits because people who typically would not have made it to clinic, we were able to have a visit by phone in their own space.”* (Clinician, Clinic 2) This was seen as particularly salient for traditionally “hard-to-reach patients,” patients living far from the clinic, and those with complex travel routes to the clinic (generally involving longer commutes on public transportation or ride requests), because*, “I think generally people miss less phone visit appointments just because it’s easier to access them, and we give them a couple tries. In-person…sometimes, people just don’t show up.”* (Clinician, Clinic 1)

Most individuals agreed that the flexibility offered by telemedicine improved the clinics’ ability to efficiently and effectively reach patients. By meeting patient’s modality preferences (i.e., in-person versus telephone versus video visit), clinicians and case managers felt better able to provide more person-centered care, with one case manager stating,* “I think anytime you provide different modes of communication, increase choice or possibilities, it increases access and increases client’s or patient choice and preference… it's overall been better [and] positive.”* (Case manager, Clinic 1) Some individuals expressed that the increased flexibility of telemedicine could lead to improved health outcomes for PLHIV (including for other chronic conditions) since time saved with telemedicine could be spent taking care of other health needs, such as specialty appointments or cancer screening appointments, expressing that, *“HIV care is not a one-size-fits-all [and] the more we can to be flexible about the options we give patients, the better off we will be in terms of seeing retention, suppression… maybe they will be more likely to go get their mammogram, or go get their colonoscopy.”* (Clinician, Clinic 2)

However, many of these stakeholders voiced concern that PLHIV face significant barriers for accessing either telephone or video telemedicine visits, particularly patients who are unhoused, have lower income levels, or lack technological resources, with some stakeholders sharing that *“Many of our patients are low-income individuals. They don’t necessarily have a computer at home that has a camera, or they don’t have a smart [phone]—they may have a cell phone, but it’s one of the old flip phones.”* (Clinic administrator, Clinic 1) One clinician stated that *“Fifty percent of my patients just flat out refuse any telemedicine, but most of those patients, again, are either homeless, in transitional housing of some form [or] they don’t have an income, so they may not have a reliable phone.”* (Clinician, Clinic 1) Many of these patients were perceived to also have insufficient technological literacy to participate in video visits. Telephone visits were generally the default modality for telemedicine (Table 4) due to clinic staff *“trying to still figure out how we're gonna do [video appointments] with a lot of our patients 'cause a lot of them are not tech savvy. Right now, we're just doing the phones.”* (Case manager, Clinic 2) One clinic administrator expressed that *“We did more telephonic visits than we did video visits because it was easier for a patient, even with an old flip phone, to actually call in and talk to a provider as opposed to actually having to do a video visit where they had to see the provider.”* (Clinic administrator, Clinic 1)

**Table 4:** Current Use of HIV Care Telemedicine and In-Person Visits by Clinicians and Case Managers

	**Overall** **(*N* = 14)** N (%)	**Clinic 1** **(*N* = 8)** N (%)	**Clinic 2** **(*N* = 6)** N (%)
**Current mode of telemedicine**			
Telephone only	9 (64)	3 (37)	6 (100)
Video only	0 (0)	0 (0)	0 (0)
Both telephone and video	5 (36)	5 (63)	0 (0)
**Percent of HIV visits that are telemedicine**^**a**^			
< 20	2 (14)	1 (12)	1 (17)
20–29	4 (29)	3 (37)	1 (17)
30–39	3 (21)	1 (12)	2 (33)
40–49	0 (0)	0 (0)	0 (0)
50 or more	3 (21)	2 (25)	1 (17)
**Preferred minimum number of in-person visits annually**^**b**^			
0	2 (14)	2 (25)	0 (0)
1	4 (29)	2 (25)	2 (33)
2	6 (43)	2 (25)	4 (67)
3 or more	1 (7)	1 (12)	0 (0)

^a^Missing data (*N*=2); ^b^Missing data (*N*=1)

Clinicians who required medical interpretation in order to have visits with monolingual Spanish-speaking PLHIV felt that interpretation done over telemedicine (either telephone or video) added additional complexity to the visit and resulted in a preference for in-person visits, with one clinician sharing*, “If I have to use a translator through the phone, it's just not the same interaction. Sometimes they like coming in, so even if I need a translator, I'm there, they can see my body language, et cetera.”* (Clinician, Clinic 2) The clinicians who were fluent in Spanish and did not require medical interpretation, however, found that Spanish-speaking PLHIV did not have a strong preference for in-person over either telephone or video telemedicine visits, affirming that, *“Language wise, I don't really get a sense that really pushes people to have one type of visit over another… I can have a lot of my visit types in Spanish… If a phone visit interaction truly is easier for [the patient] versus a face-to-face visit, they'll let me know.” (*Clinician, Clinic 1)

#### Telemedicine implementation: challenges and solutions

While the adaptability of telemedicine created advantages for PLHIV, the abrupt need to initiate telemedicine due to SARS-CoV-2 exacerbated the implementation challenges faced by clinics. These challenges included creating workflows to incorporate telephone and video telemedicine into visit protocols and adding new responsibilities to staff roles, because *“Telehealth requires an added level of pre- and post-visit planning… We need to do the same things in the virtual world that we've been doing in the physical world, which is having a staff member available to room the patient, but they take more time and more coordination, and there's more opportunity for delay.”* (Policymaker) One clinician shared that* “It’s just been cumbersome to tie in the medical assistants [MAs] into those video visits too where, in a normal first face-to-face visit, they would usually be seeing the patient, do the vital signs, checking them in and doing their chief complaint, updating their medication list, et cetera.”* (Clinician, Clinic 1)

Balancing schedules that included both in-person and telemedicine visits (either telephone or video) was also challenging for clinic staff, but many found solutions over time, as discussed by one case manager who felt that* “It was challenging in the beginning because we were learning as we go, and organizing the schedule was a work in progress… We have gotten to this stage where we would dedicate the first portion of the clinic time for in-person visits, and then the remainder of the clinic time would be for telemedicine visits.”* (Case manager, Clinic 2) Although both facilities incorporated phone visits quickly and with overall success, respondents cited barriers with video visits due to difficulties with video visit platforms and lack of staff buy-in, with one clinic administrator stating, *“We've actually switched multiple times and tried different [video visit] platforms that would be easier to use for patients and easier to use for staff… The third interface was the easiest, but… it was harder to get the buy-in because at this point, they had gotten used to doing the phone calls.” (*Clinic administrator, Clinic 1)

Some Clinic 1 staff also perceived limited leadership buy-in to telemedicine, mentioning *“Our main medical leadership wasn't pushing [telemedicine]… [and] was also leery about it and not really 100 percent about it and, in my honest opinion, probably didn't really even wanna do it.”* (Clinic administrator, Clinic 1) In contrast, Clinic 2 staff stated significant support during the telemedicine implementation process, where *“The leadership has been onboard as well… Me personally, I didn’t really feel that there [was] pushback or a deer-in-the-headlights scenario… In fact, there are even non-clinical people who wanted to know and learn how can they be of help in promoting telehealth. I think this was really like a—it was a good team effort.”* (Clinic administrator, Clinic 2)

#### Telemedicine training

Clinicians and case managers reported variation in their telemedicine training. A few completed an initial training that they felt was adequate for their needs, but many shared that they had never been trained on how to use telemedicine (either telephone or video) nor how to incorporate it into their workflow. Nonetheless, individuals generally felt like they could access information and additional training if needed, with one clinician sharing, *“I know that there were several afternoons blocked where they did training with the electronic medical record. I’m not in clinic every day so I wasn’t there for one of the trainings, so I didn’t do it… I probably could be more aggressive about seeking out training on the video.”* (Clinician, Clinic 2)

Even with no additional training, most individuals were confident in their capacity to provide quality care to their patients over telephone visits. Some also shared that, as they did more telephone visits over time, their skills and comfort increased: *“In* *the beginning, [telemedicine] was pretty daunting, but I think over the first few months, both my patients and I got more comfortable with it. I think it's actually been the preferred method of interaction between more and more of my patients.”* (Clinician, Clinic 1)

#### Beliefs about telemedicine

Individuals interviewed exhibited diverse opinions about telemedicine for HIV care. Many had positive attitudes and liked using telemedicine for routine visits, such as one clinician who stated that *"...[it] was very much needed that we started expanding doing [telemedicine], and it’s becoming more accepted. It’s something we can do as a more routine type of visit. I’m very satisfied that we have expanded that type of care.”* (Clinician, Clinic 1) In some cases, this positive attitude was based, in part, on their own use of telemedicine for their personal health, with one clinic administrator sharing, *“I’ve had telehealth visits with my provider, and it’s actually cool. I’d almost prefer it. That way, I don’t have to get up and go into the clinic.”* (Clinic administrator, Clinic 1) In contrast, some interviewees had negative views of telemedicine (both telephone and video) for HIV care, citing reasons such as patients being generally unhappy with the quality of care provided over telemedicine and preferring in-person visits. One clinician expressed, *“I might have already revealed my bias… I hate it. I hate it because I don't like telehealth. I hate it because we end up with wasted time slots…'cause patients would rather come in person than do telehealth.”* (Clinician, Clinic 1)

With regard to telemedicine use for behavioral care and case management purposes, clinicians found this to be generally suitable for PLHIV. They reported that, for mental health, people *“seem to do quite well with telehealth [and] it seems to be quite popular for the providers and the patients.”* (Clinician, Clinic 1) This was also true for case management: *“Folks that require lots of care and continuous follow up really benefit from being able to access us through technology.”* (Case manager, Clinic 1)

Clinical, programmatic, and policy stakeholders agreed that in-person care for PLHIV who were newly diagnosed was and should remain the norm, as detailed by one clinician who stated, *“It’s pretty unusual for us to do phone visit for [new diagnoses]… I think all those nonverbal cues are really important, especially with a new diagnosis, having a discussion, education about what that means, and giving support to the patient, getting their history. I think [in-person care] establishes that rapport, which for a new HIV diagnosis, that’s very, very important to make sure they stay engaged in care.”* (Clinician, Clinic 1)

## Discussion

Our study showed that despite being a new mode of HIV care delivery for these two FQHCs, most stakeholders found telemedicine to be highly acceptable.

PLHIV participants with exposure to telemedicine were overwhelmingly interested in continuing telemedicine long-term. Our findings build on those of previous studies, which have shown that patient satisfaction with telemedicine for primary care is similar to in-person visit satisfaction [20, 21]. Our study revealed that telemedicine provides a number of important benefits for PHLIV, including saving time (commuting and waiting at the clinic) and money (transportation costs and/or due to lost wages). These findings echo those found in the literature – telemedicine reduces the burden of complex, expensive commutes and demanding work schedules and reduces stress stemming from travel [10].

Stakeholders agreed about telemedicine in several areas (Table 5): that a new HIV diagnosis was better managed in person and that telemedicine reduces time spent and costs for PLHIV, decreases missed and late appointments, and is good for behavioral care and case management. However, there were also several areas where perspectives and opinions diverged. Almost all PLHIV felt satisfied with the quality of care they received during their telemedicine (predominantly telephone) visits, desired telemedicine visits as part of their care, and did not feel that their relationships with clinicians were compromised. Many clinicians, on the other hand, perceived that PLHIV preferred in-person visits over telemedicine due to perceived higher quality of care and for better rapport. Also, while the majority of clinical, programmatic, and policy stakeholders raised concerns about patients’ access to technology resources and technology literacy, these barriers were raised only by a minority of PLHIV. Spanish-speaking PLHIV also found interpretation over telephone telemedicine to be acceptable even though some clinicians felt it was a challenge and did not work well.

**Table 5:** Comparison of Telemedicine Benefits and Challenges as reported by PLHIV, Clinical, Programmatic, and Policy Stakeholders

*Benefit / Challenge*	*People Living with HIV (PLHIV)*	*Clinical, Programmatic, and* *Policy Stakeholders*
*Acceptability*	All but one interested in continuing to use telemedicine in their HIV care	Perception that PLHIV prefer in-person visits
*Travel to clinic and opportunity costs*	Less travel time, saves time and costs of transportation for PLHIV Fewer missed/late appointments for PLHIV Reduced number of visits, especially for stable and suppressed PLHIV, that gives more time for work and personal matters	
*Technological literacy*	Some prefer telephone, some prefer video Most report feeling tech literate for telephone telemedicine or are interested in learning more about how to use video	Perception that most PLHIV prefer telephone because it is simpler to use
*Technology resources*	Most reported access to smartphone, computer/tablet and Wi-Fi/mobile data	Perception that Wi-Fi/mobile data not dependable for many PLHIV Perception that PLHIV with housing instability/homelessness may not have consistent access to telephone or computer/tablet
*New diagnoses*	Better for initial visits to be in person	
*Privacy*	Many reported access to privacy for telemedicine visits and most are comfortable with being on video	Concerns around patients’ privacy at home/in surrounding environment during visit
*Spanish interpretation*	Use of interpreters over telemedicine is acceptable	Use of interpreters over telemedicine more difficult

Privacy can be an important barrier to telemedicine care, particularly for individuals cared for in FQHC settings. Despite this concern, we found that many participants in our study reported having access to private space. Those who did not have private space did report this as a barrier to both telephone and video telemedicine for their HIV care. This echoes previous findings from the literature about the important role that privacy plays in the likelihood of PLHIV using telemedicine [22]. Because living circumstances are dynamic, it is important for clinicians to avoid assumptions about privacy and to routinely offer telemedicine for all interested patients, as they may experience periods of time where they may have access to private spaces for telephone or video visits.

There was a high degree of individual-level variation in clinicians’ perceptions of telemedicine. This finding suggests that leadership will need to work closely with clinicians to explore perceptions and potential biases and ensure that all patients are given equitable access and clinicians are supported to overcome barriers and challenges with the use of telemedicine. We also found important clinic-level differences in our data, with Clinic 1 having limited perceived leadership buy-in and staff commonly having negative views of both telephone and video telemedicine and reluctance to engage in telemedicine; whereas Clinic 2 staff reported high levels of acceptance in the setting of perceived leadership enthusiasm and support for telemedicine. These data suggest that perceived leadership buy-in can affect the culture of a clinic and the subsequent implementation of telemedicine, and the literature has shown that these factors are responsible for impacting the implementation of all types of health service delivery [23, 24]. Studies suggest that exploring and addressing clinician experiences, beliefs, and biases is crucial to creating an environment that is open to implementing and scaling new interventions like telemedicine [25, 26]. This can be done through continuing medical education training that focuses on the relevant patient- and clinic-level benefits of telemedicine, as well as the evidence behind the new intervention [26]. The provision of evaluation data showing high-level patient acceptability and feasibility with regard to the availability of technology and privacy may also help staff feel motivated to continue to provide telemedicine.

The use of telephone visits has been unique to the SARS-CoV-2 pandemic. Telephone visits became reimbursable given the urgent need to ensure the safety of clinic staff and patients and comply with widespread stay-at-home orders in 2020. While telemedicine modality options for PLHIV included, in theory, both telephone and video visits, most of our interviewees had not yet been offered or used video visits due in large part to technical challenges at the two clinics, including difficulties with finding and implementing functional video visit platforms, lack of workflows to support clinicians performing video visits, and decreased buy-in from staff. The trend of telephone visits being the primary modality for telemedicine aligns with what has happened in California and across the U.S. during the rapid expansion of telemedicine during the pandemic [27, 28]. In a Ryan White-funded HIV clinic in Seattle, Washington, telemedicine (telephone and video) increased from less than 1% of all visits pre-pandemic to 80% early in the pandemic, with 52% of visits occurring via telephone in April 2022 [29]. A similar trend was seen for the use of telemedicine for primary and behavioral health care among safety-net organizations in the state of California in March 2020, with 48.5% of all visits occurring via telephone and only 3% by video [27].

Of PLHIV in our study with a preference for a certain modality of telemedicine, approximately half preferred telephone and half video. Individuals largely spoke about video from a theoretical perspective since few had experience with this modality for their health care. Some participants had experience with FaceTime or similar videochatting technologies and felt comfortable with smartphones and use of apps, suggesting that these individuals would have adequate technological literacy for telemedicine with video, if this were to be offered. Some PLHIV expressed openness to using video, but felt they needed training to do so. Literature suggests that even a small amount of education around telemedicine with video empowers patients to feel more confident in their technological abilities [22] and can lead to greater interest and use of video for their care [30]. If insurance reimbursement for telephone visits ends as SARS-CoV-2 enters an endemic phase, video could be the only modality for patients at FQHCs to access telemedicine. Additionally, while the use of telephone visits has been an important strategy for keeping people engaged in HIV care in the near-term, lack of access to video visits for patients of FQHCs may contribute to widening health inequities [28]. Creating video opportunities for historically marginalized patients, such as those with less access to devices and fewer opportunities for formal technological education, those who are not English-dominant, and with lower incomes, allows for a better person-centered approach. If patients are given modality options for their care that can be tailored to their preferences and needs (instead of these preferences being assumed and their options being restricted from the outset), this may help achieve the greater goal of more equitable HIV care.

One potential solution to support expansion of video includes the use of “Telemedicine Navigators” – individuals embedded in clinics and tasked with teaching patients to connect to a video visit and assisting with any technical support issues leading up to, or at the time of, the visit [12, 31]. Additional funding will be needed to support navigators and research on costing and cost-outcome data may be helpful for health system and clinic leaders as they consider this option.

An important theme from the majority of interviews was the flexibility provided by being able to use a combination of telemedicine (either telephone or video) and in-person care. PLHIV with exposure to telemedicine largely preferred a 50/50 mix of in-person and telemedicine visits, while some preferred to receive all of their care via telemedicine. Only one person desired all in-person care. Clinicians’ preferred minimum number of in-person visits per year for PLHIV who were stable and virologically suppressed was two (or approximately every six months), with some variation based on the clinical scenario and individual patient needs. These data suggest that the approach to telemedicine must be tailored to the patient interest as well as the clinical scenario and need for in-person care, such as acute illness requiring physical exam, vaccinations, blood sugar and/or blood pressure checks (if remote monitoring programs are unavailable), and/or the need for annual preventive visits and screens (e.g. physical exam, cervical cancer screening, etc.). Ultimately, the incorporation of telemedicine was viewed by clinical, programmatic and policy stakeholders as an opportunity to improve PLHIV’s engagement and decision-making in their own care, contributing to a more person-centered approach [32].

### Limitations

Our study has several limitations. Due to the SARS-CoV-2 pandemic, all interviews were conducted over the telephone or via Zoom, which biased the sample towards inclusion of PLHIV facing fewer challenges with connecting via these modalities. Therefore, our study does not represent the perspectives of those who might have the largest barriers to telemedicine. Additionally, as mentioned above, the telemedicine programs at both clinics almost entirely consisted of telephone visits, so discussion about the use of video was largely theoretical and not based on actual experience. Our study may be limited by the fact that client-facing stakeholders (clinicians and case managers) were speaking in relation to their complete experience with clients, while PLHIV represented only a small sample of each clinic’s population. However, participants at Clinic 2 were broadly representative of the overall clinic population (with regard to age, gender, housing status, and race/ethnicity), while participants at Clinic 1 were similar with the exception of overrepresentation of women (33% in our sample versus 12% overall) and Hispanic/Latino PLHIV (60% in our sample versus 25% overall [includes multi-racial Hispanic/Latino PLHIV]) and underrepresentation of Black and African-American PLHIV (13% in our sample versus 40% in the clinic overall [includes multi-racial Black and African-American PLHIV]). Our study also included only two FQHCs in the same region of south Los Angeles County. Given that the views of stakeholders will likely vary in different types of clinical settings and in different regions of the United States, future studies should explore a range of care settings with diverse geographic contexts, particularly those that consider issues like distance to care (rural versus urban) and accessibility (availability of reliable and affordable public transportation, role of traffic in ease of accessing care, etc.) [33]. Finally, we were limited in making comparisons between professional roles given that our sample size within each group was too small.

## Conclusions

Telemedicine, delivered overwhelmingly as telephone visits in two FQHCs in Los Angeles during the early SARS-CoV-2 pandemic, alleviated barriers to HIV care for PLHIV, such as time and cost of travel. PLHIV in our study found telemedicine highly acceptable and feasible and expressed overwhelming interest in continuing telemedicine visits long-term, including some who desired access to video visits. While clinical, programmatic and policy stakeholders raised concerns about patient challenges with access to technology and privacy for telemedicine (concerns not shared by most PLHIV clients), they valued the benefits for patients and the flexibility of adapting the visit type to patient preference, which was also viewed as an advancement in the ability to provide person-centered care. Future research should focus on best practices for the use of video and exploring strategies to help patients and clinics overcome barriers to ensure equitable access to telemedicine. Research is also needed to understand whether telemedicine can improve HIV outcomes, including sustained engagement in care and viral suppression.

## Data Availability

Data and materials used for this study are available upon request and approval by the corresponding author.

## Abbreviations

ART: Antiretroviral therapy
CMS: Centers for Medicare & Medicaid Services
FQHC: Federally qualified health center
HIV: Human immunodeficiency virus
IDI: In-depth interview
PLHIV: People living with HIV
RW: Ryan White
SARS-CoV-2: Severe acute respiratory syndrome coronavirus 2
SPA: Service Planning Area
U.S.: United States
